# Chemical complementarity of tumor resident, T-cell receptor CDR3s and renalase-1 correlates with increased melanoma survival

**DOI:** 10.18632/oncotarget.28633

**Published:** 2024-08-05

**Authors:** Saif Zaman, Fred S. Gorelick, Andrea Chrobrutskiy, Boris I. Chobrutskiy, Gary V. Desir, George Blanck

**Affiliations:** ^1^Department of Internal Medicine, Yale School of Medicine, New Haven, CT 06510, USA; ^2^Veteran’s Administration Healthcare System, CT 06516, USA; ^3^Department of Cell Biology, Yale School of Medicine, New Haven, CT 06511, USA; ^4^Department of Pediatrics, Oregon Health and Science University Hospital, Portland, OR 97239, USA; ^5^Department of Internal Medicine, Oregon Health and Science University Hospital, Portland, OR 97239, USA; ^6^Department of Molecular Medicine, Morsani College of Medicine, University of South Florida, FL 33612, USA; ^7^Department of Immunology, H. Lee Moffitt Cancer Center and Research Institute, Tampa, FL 33612, USA

**Keywords:** RNLS, melanoma, T-cell receptor CDR3s, chemical complementarity

## Abstract

Overexpression of the secretory protein renalase-1 negatively impacts the survival of melanoma and pancreatic cancer patients, while inhibition of renalase-1 signaling drives tumor rejection by promoting T-cell activation. Thus, we investigated the chemical complementarity between melanoma-resident, T-cell receptor (TCR) complementarity-determining region 3 (CDR3) amino acid sequences (AAs) and the renalase-1 protein. Increasing complementarity of TCR CDR3s to renalase-1 AAs, as assessed by a chemical complementarity scoring algorithm, was associated with improved overall survival (OS) in melanoma patients. The expression levels of several immune signature genes were significantly, positively correlated with increasing TCR CDR3-renalase-1 complementarity scores. Additionally, the survival association observed with high complementarity of TCR CDR3s to renalase-1 AAs was more robust in cases with low renalase-1 gene expression levels. Mapping of TCR CDR3-renalase-1 *in silico* interaction sites identified major epitope candidates including RP220, the signaling module of the renalase-1 protein, consistent with the fact that a monoclonal antibody to RP220 is a potent inhibitor of melanoma growth. These findings indicate that renalase-1 is a potential antigen for TCR recognition in melanoma and could be considered as a target for immunotherapy.

## INTRODUCTION

Increased tumor levels of the protein encoded by the renalase gene (RNLS) have been associated with decreased survival in pancreatic adenocarcinoma and melanoma [1, 2]. In patients with melanoma treated with either anti-PD1 or anti-CTLA4 or both, increased tumor RNLS levels were associated with a worse outcome [3]. The RNLS protein in these cancers represents the splice variant referenced as “Renalase-1” [4], which in this report is hereinafter referred to as the RNLS protein. In prior experiments, inhibition of RNLS expression in melanoma decreases tumor cell proliferation and leads to more effective immune responses. Specifically, Guo et al. [3] demonstrated that RNLS knock-out results in T-cell-dependent melanoma tumor regression in mice. Additionally, inhibitory antibodies raised against the RP220 signaling module of the RNLS protein reduce RNLS tumor expression and tumor growth in murine melanoma models when given as single agents [2]. The anti-RP220 antibodies also synergize with anti-PD-1 [3]. These results suggest that inhibition of RNLS protein signaling in a host can change the tumor microenvironment and lead to tumor-directed, T-cell-mediated cytotoxicity [3].

The availability of large-scale sequencing data for human tumors has made the assessment of intratumor, patient-specific, adaptive immune receptor recombination events highly feasible. For example, our group has demonstrated that chemical complementarity between tumor resident, T-cell receptor, complementarity-determining region 3 (CDR3s), and MAGEA3/6 correlates with increased survival in patients with melanoma [5]. Given our data regarding (i) anti-RNLS protein antibodies reducing melanoma tumor growth *in vivo* using mouse models and (ii) *in silico* approaches to assess patient-specific T-cell receptor CDR3s from human tumor samples, we hypothesized that T-cell receptor-RNLS AA sequence chemical complementarities would associate with improved survival probabilities.

## RESULTS

### Increasing complementarity of TCR CDR3s to RNLS AAs is associated with improved OS outcomes

**Figure 1:**
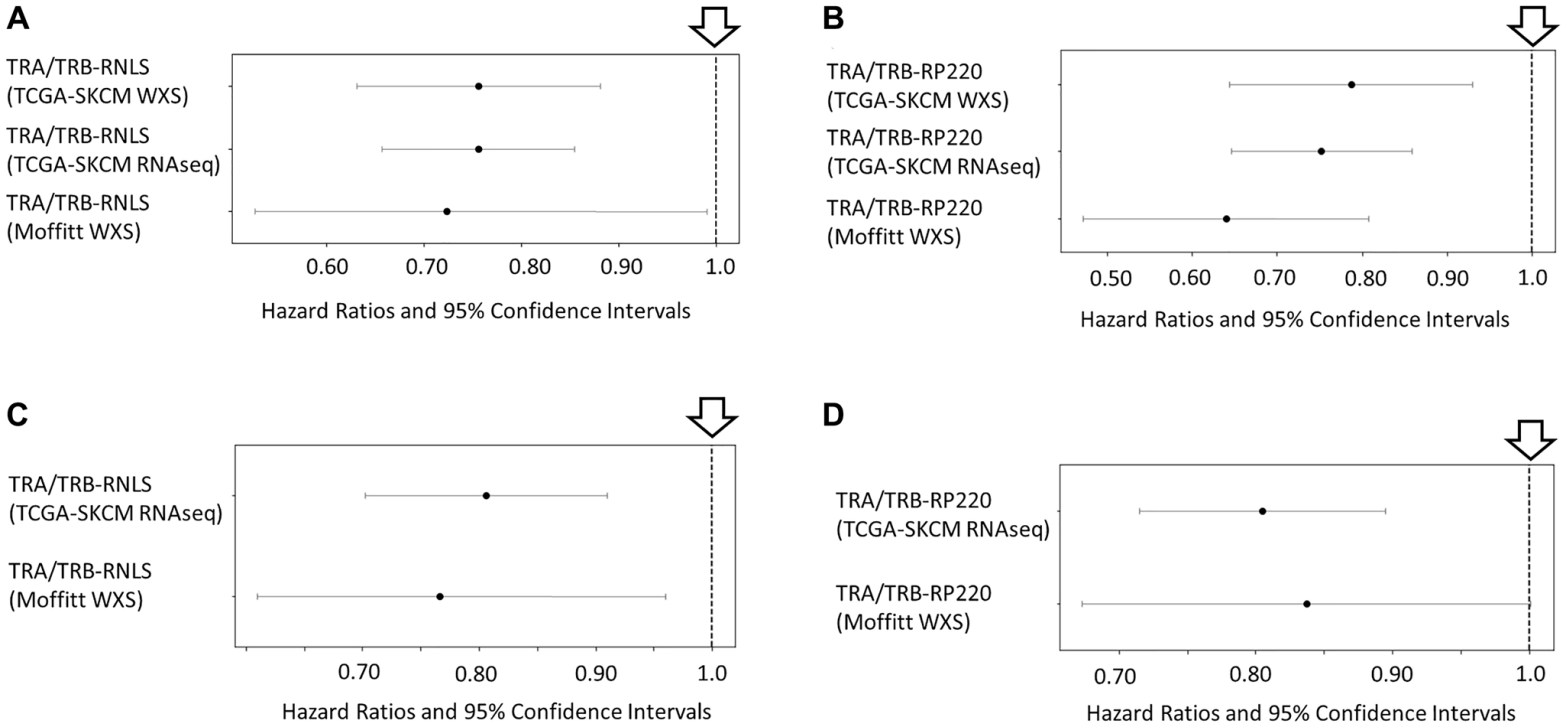
Overall survival (OS) associations with TCR complementarity to RNLS AAs and the renalase peptide RP220. Using a univariate Cox analysis, Forest plots comparing the OS probability results based on Electrostatic CSs for the TCR (TRA +TRB) and RNLS AAs or the RNLS RP220 peptide. Black circles on the horizontal line represent the point estimate of the hazard ratio (HR) for a given sample. The genomics file sources of the TCR recombination reads and the TRA and TRB CDR3 AA sequences are indicated (TCGA-SKCM WXS, TCGA-SKCM RNAseq, Moffitt WXS), with the length of the line corresponding to the width of the 95% confidence interval (CI). The vertical line at the null value of OR = 1 (downward arrow) indicates no association between the TCR CDR3 CS to RNLS AA sequences and the OS probability. (**A**) Improved OS probability associated with increased Electrostatic CSs based on TCR CDR3s and RNLS, with the CDR3s obtained from the TCGA-SKCM WXS files (HR = 0.756, CI = (0.631, 0.906), *p* = 0.0024; CDR3s obtained from TCGA-SKCM RNAseq files (HR = 0.756, CI = (0.657, 0.87), *p* < 0.001); CDR3s obtained from the Moffitt WXS files (HR = 0.724, CI = (0.527, 0.995), *p* = 0.046). (**B**) Improved OS probability associated with increased Electrostatic CSs based on TCR CDR3s and RP220, with CDR3s obtained from the TCGA-SKCM WXS files (HR = 0.787, CI = (0.644, 0.963), *p* = 0.019); from the TCGA-SKCM RNAseq files (HR = 0.752, CI = (0.646, 0.874), *p* = 0.00021); and from the Moffitt WXS files (HR = 0.640, CI = (0.472, 0.868), *p* = 0.0041). (**C**) Improved OS probability associated with increased Combo CSs based on TCR CDR3s and RNLS, using the CDR3s recovered from the TCGA-SKCM RNAseq files (HR = 0.806, CI = (0.702, 0.925), *p* = 0.0022); and from the Moffitt WXS files (HR = 0.766, CI = (0.610, 0.962), *p* = 0.021). (**D**) Improved OS probability associated with increased Combo CSs based on TCR CDR3s and RP220, using CDR3s from the TCGA-SKCM RNAseq files (HR = 0.805, CI = (0.715, 0.906), *p* = 0.0003). No significant association with OS probability when assessing Moffitt WXS (HR = 0.837, CI = (0.673, 1.04), *p* = 0.11).

Univariate Cox proportional hazard models for OS associations with TCR (TRA + TRB) CDR3 AA complementarity to RNLS AAs were assessed for human melanoma tumor samples (Methods). With higher electrostatic complementarity between TCR CDR3s and the RNLS AA sequence, there was significantly improved OS probability based on CDR3 AA sequences recovered from (i) the TCGA-SKCM WXS files, (ii) the TCGA-SKCM RNAseq files, and (iii) the Moffitt Cancer Center WXS files (Figure 1A). (Statistical summary: TCGA-SKCM WXS-recovered CDR3s (HR = 0.756, CI = (0.631, 0.906), *p* = 0.0024; TCGA-SKCM RNAseq-recovered CDR3s (HR = 0.756, CI = (0.657, 0.87), *p* < 0.001); Moffitt Cancer Center WXS-recovered CDR3s (HR = 0.724, CI = (0.527, 0.995), *p* = 0.046). Note that the TCGA-SKCM RNAseq-recovered CDR3s were provided by an independent research group [6].

Given observations that monoclonal antibodies raised against a strongly evolutionarily conserved domain of the RNLS protein, the RP220 peptide, are potent inhibitors of cancer cell proliferation [2, 3], we assessed the potential for RP220 to be an antigenic target for T cells, using the above bioinformatics (*in silico*), CDR3 chemical complementarity scoring approaches. When assessing electrostatic complementarity between TCR CDR3s and the RP220 peptide of RNLS, there was a significantly improved OS probability associated with the higher CSs, specifically when assessing TCR CDR3s represented by the (i) TCGA-SKCM WXS files, (ii) the TCGA-SKCM RNAseq files, and (iii) the Moffitt Cancer Center WXS files (Figure 1B). (Statistical summary: TCGA-SKCM WXS-recovered CDR3s (HR = 0.787, CI = (0.644, 0.963), *p* = 0.019); TCGA-SKCM RNAseq-recovered CDR3s (HR = 0.752, CI = (0.646, 0.874), *p* = 0.00021); Moffitt WXS-recovered CDR3s (HR = 0.640, CI = (0.472, 0.868), *p* = 0.0041).

To assess whether hydrophobic forces could influence TCR CDR3-candidate antigen interaction, we assessed potential OS probability distinctions with the use of a “Combo CS” that added a value (to the electrostatic calculations) based on Uversky hydropathy values for each AA [7]. The Combo CSs for the TCR CDR3-RNLS AA pairs provided significantly improved OS probability distinctions when assaying the CDR3s representing cases provided by the Moffitt Cancer Center tumor WXS files, and when assaying the CDR3s recovered from the TCGA-SKCM RNAseq files (Figure 1C). (Statistical summary: Moffitt WXS-recovered (HR = 0.766, CI = (0.610, 0.962), *p* = 0.021), TCGA-SKCM RNAseq-recovered (HR = 0.806, CI = (0.702, 0.925), *p* = 0.0022). The Combo CS for the TCR CDR3-RP220 peptide representing CDR3s from the TCGA-SKCM RNAseq files also represented an OS probability distinction (Figure 1D; HR = 0.805, CI = (0.715, 0.906), *p* = 0.0003). No significant association with OS probability was observed when assessing the RP220 Combo CSs using the Moffitt Cancer Center WXS-recovered CDR3s (HR = 0.837, CI = (0.673, 1.04), *p* = 0.11).

### Gene expression analysis

**Figure 2:**
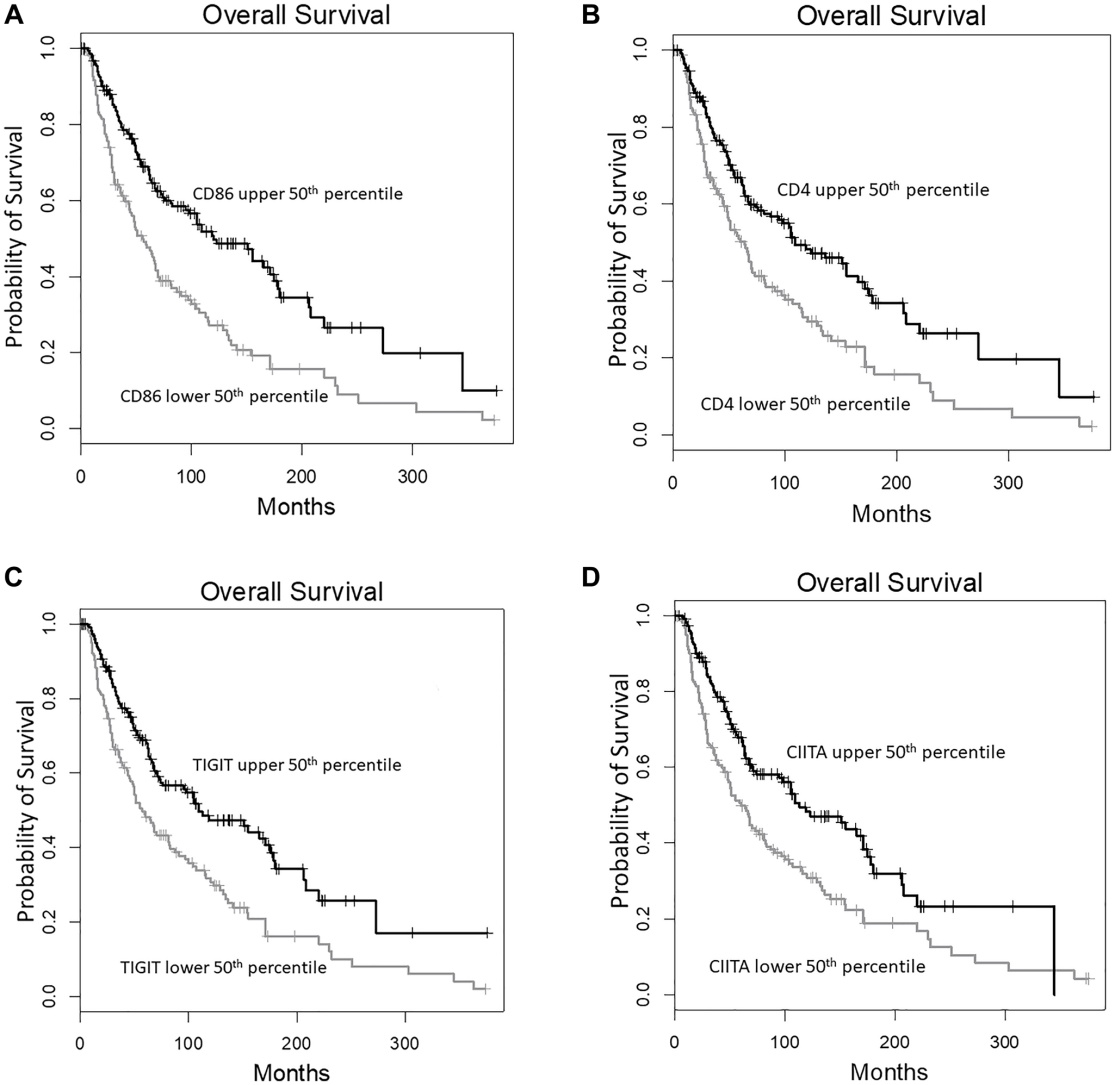
Immune signature gene expression levels correlated with high TCR CDR3-RNLS CSs are independently associated with a high OS probability. KM survival analysis, assessing the four genes from Table 2 with the greatest, statistically significant correlations with TCR CDR3-RNLS CSs. In this KM analyses, the OS probability for cases representing the upper 50th percentile of the RNAseq values (black) is compared to cases representing the lower 50th percentiles of the RNAseq values (grey). The immune signature genes representing the KM analyses are displayed as follows: (**A**) CD86 (upper 50th percentile, *n* = 229; lower 50th percentile, *n* = 229; Log rank *p* = 9.3E-7; Hazard ratio = 0.52) (**B**) CD4 (upper 50th percentile, *n* = 229; lower 50th percentile, *n* = 229; Log rank *p* = 0.00013; Hazard ratio = 0.59) (**C**) TIGIT (upper 50th percentile, *n* = 229; lower 50th percentile, *n* = 228; Log rank *p* = 2.1E-5; Hazard ratio = 0.56) (**D**) CIITA (upper 50th percentile, *n* = 229; lower 50th percentile, *n* = 229; Log rank *p* = 2.7E-5; Hazard ratio = 0.59).

To assess whether Electrostatic CSs for the TCR CDR3-RNLS AA sequence alignment pairs correlated with gene expression levels for an immune signature gene panel [8], we generated scatterplots based on the CSs calculated for the CDR3s obtained from the tumor WXS and RNA-seq files, respectively. First, using the SKCM WXS-recovered TCR CDR3s, we found that the expression of 27 of 51 immune signature genes significantly correlated with the Electrostatic CSs, after a Bonferroni correction (*p* < 0.000098) (Table 1). Next, using the SKCM RNAseq-recovered TCR CDR3s, we found that the expression of 40 of 51 immune signature genes significantly correlated with the Electrostatic CS representing the TCR CDR3-RNLS AA sequence pairs after a Bonferroni correction (*p* < 0.000098) (Table 2). Histograms with Pearson’s correlation coefficients for the association of gene expression levels and Electrostatic CSs for the indicated, expressed genes are shown in Supplementary Figure 1A, 1B. Finally, with reference to the potential of this approach to identify independent biomarkers, we investigated whether the expression of immune signature genes correlated with the Electrostatic CSs (Table 2) could be biomarkers independently of the CSs. We found that elevated expression levels of CD86, TIGIT, CIITA, and CD4, representing the four genes whose expression most significantly correlated with the Electrostatic CS, as indicated in Table 2, were significantly associated with improved OS probabilities (Figure 2).

**Table 1:** Pearson’s correlation coefficients for the correlation of the expression of immune signature genes with Electrostatic CSs, based on RNLS AA sequences and the CDR3s recovered from TCGA-SKCM-WXS files

Gene	Pearson correlation coefficient	*p*-value
TIGIT	0.35888785	2.14E-10
CD3D	0.35534465	3.31E-10
CIITA	0.34322386	1.40E-09
CD226	0.33369743	4.19E-09
CD86	0.33025592	6.17E-09
CD4	0.32030109	1.83E-08
PDCD1	0.32011279	1.87E-08
PVRIG	0.31503371	3.21E-08
CD8A	0.31408719	3.55E-08
ICOS	0.31051577	5.16E-08
CD38	0.29578225	2.28E-07
CD72	0.29515825	2.42E-07
GZMA	0.29423052	2.65E-07
IFNG	0.28393199	7.11E-07
LAG3	0.28040759	9.87E-07
PRF1	0.27716274	1.33E-06
TNFRSF9	0.27616769	1.46E-06
BTK	0.25021878	1.37E-05
TCF7	0.24148023	2.76E-05
CXCL12	0.24105594	2.86E-05
CD33	0.23931222	3.28E-05
CXCL13	0.23598787	4.24E-05
ITGAX	0.23458702	4.72E-05
CD83	0.21786675	0.00016227
TNFRSF17	0.20961476	0.0002886
GZMB	0.1982226	0.00061668
CD79A	0.19229115	0.00090107

27 of 51 genes assayed have significant positive Pearson’s correlation coefficients when using Bonferroni correction (*p* < 0.00098).

**Table 2:** Pearson’s correlation coefficients for the correlation of the expression of immune signature genes with Electrostatic CSs, based on RNLS AA sequences and the CDR3s recovered from TCGA-SKCM-RNAseq files

Gene	Pearson correlation coefficient	*p*-value
CD86	0.51464335	1.45E-28
CD4	0.4779666	2.46E-24
TIGIT	0.4773563	2.86E-24
CIITA	0.47305739	8.29E-24
CD3D	0.46630513	4.28E-23
TNFRSF9	0.46472016	6.26E-23
CD226	0.45701446	3.86E-22
ICOS	0.45632452	4.54E-22
CD72	0.43794181	2.88E-20
CD38	0.43644618	3.99E-20
PDCD1	0.41984159	1.35E-18
GZMA	0.41257412	5.93E-18
PVRIG	0.40753195	1.62E-17
CD8A	0.40502561	2.66E-17
BTK	0.38599998	9.87E-16
CXCL12	0.36929244	1.96E-14
PRF1	0.36557165	3.72E-14
LAG3	0.35265501	3.24E-13
IFNG	0.34366048	1.38E-12
TNFRSF18	0.33844769	3.14E-12
GZMB	0.33008873	1.13E-11
PDCD1LG2	0.31475057	1.08E-10
TNFRSF17	0.31421899	1.16E-10
CD33	0.31322392	1.34E-10
CTSC	0.31193419	1.61E-10
CXCL13	0.30865016	2.55E-10
CD79A	0.28688127	4.70E-09
ITGAX	0.27885756	1.29E-08
TNFRSF13B	0.26433157	7.48E-08
CD68	0.25470091	2.26E-07
TCF7	0.25314337	2.69E-07
CD19	0.24904268	4.24E-07
CD79B	0.2490033	4.26E-07
FCGR2B	0.23510083	1.88E-06
CD83	0.22837243	3.73E-06
MS4A1	0.22207123	6.96E-06
TNFRSF13C	0.2185768	9.76E-06
CD22	0.20546429	3.31E-05
CD274	0.19394521	9.09E-05
CR2	0.17927347	0.00030321
PVR	–0.1291832	0.00951641

40 of 51 genes have significant positive Pearson’s correlation coefficients when using Bonferroni correction (*p* < 0.00098).

### Electrostatic CSs for TCR CDR3-RNLS AA sequences have a more robust association with OS probabilities for cases with low expression of RNLS

**Figure 3:**
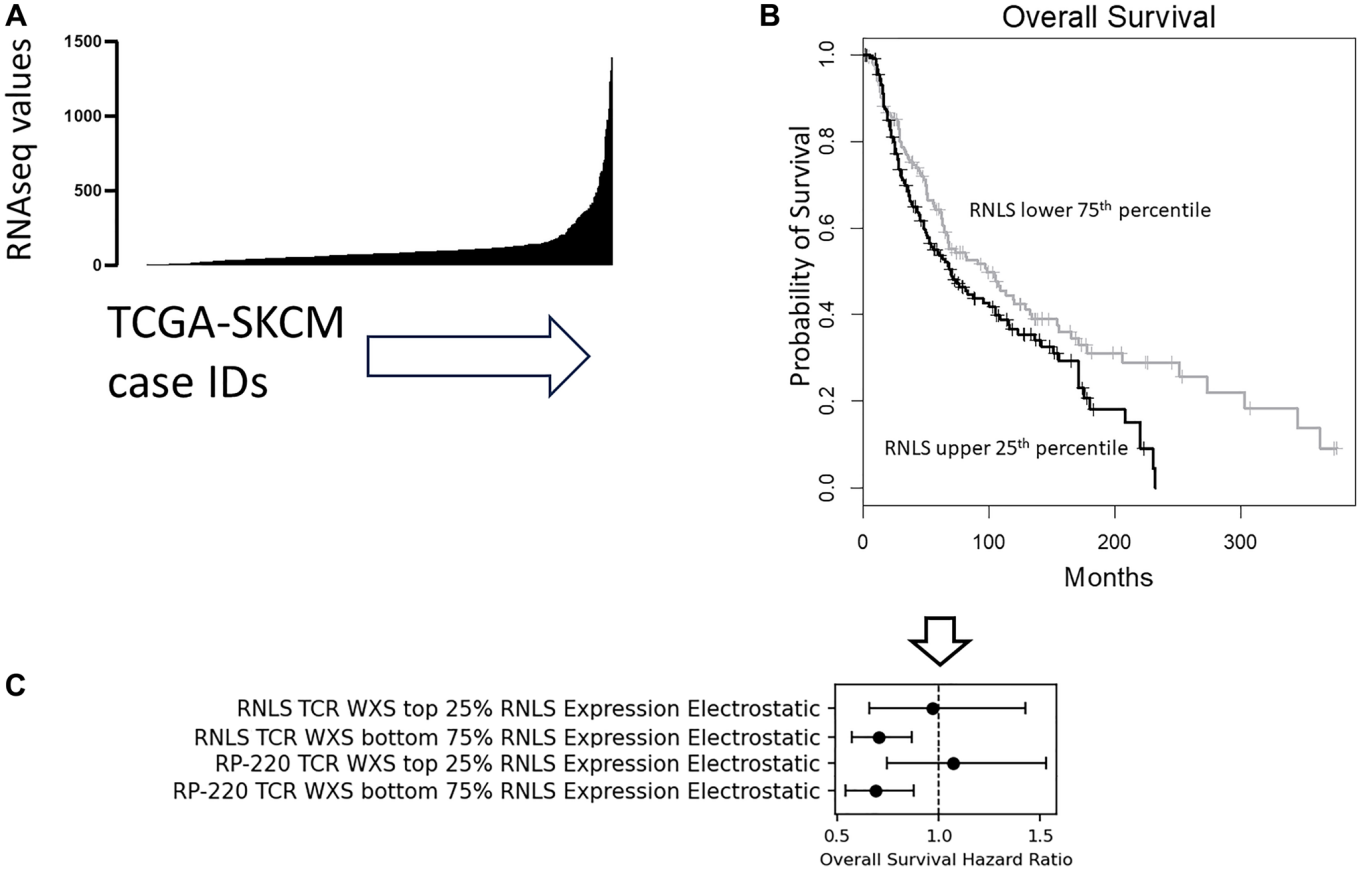
TCR CDR3-RNLS Electrostatic CSs better reflect a higher OS probability when the RNLS expression is low. (**A**) Histogram showing the distribution of RNLS RNAseq values for 443 TCGA-SKCM cases. The X-axis represents number of cases, and the Y-axis represents the RNAseq values (Methods) for RNLS. The first quartile (Q1), median, and third quartile (Q3) RNLS RNAseq values are 43.23, 79.45, and 117.15, respectively. (**B**) KM survival analysis, comparing OS probabilities for cases representing the upper 25th percentile (black line) of RNLS RNAseq values to cases representing the lower 75th percentile (grey line) of RNAseq values (upper 25th percentile, *n* = 107; lower 75th percentile, *n* = 318; Log rank *p* = 0.023). (**C**) Using a univariate Cox analysis, Forest plots comparing the OS probability results based on the Electrostatic CSs for TCR (TRA + TRB) CDR3s and RNLS or RP220, as indicated in the figure. Black dots on the horizontal line represent the point estimate of the hazard ratio (HR) for the indicated cohort of TCGA-SKCM cases, with the TCR CDR3s used to calculate the Electrostatic CSs obtained from the translation of recombination reads recovered from tumor WXS files. Results for cases representing the upper 25th percentile of RNLS RNAseq values and results for the cases representing the lower 75% of RNLS RNAseq values are indicated. The length of the black line in the figure corresponds to the range of the 95% confidence interval (CI). The vertical line at the null value of OR = 1 (arrow) indicates no association between the TCR CDR3-RNLS Electrostatic CSs and the OS outcome, if the CI line crosses that vertical indicator. When generating the Electrostatic CSs with the RNLS AA sequence, for cases representing the upper 25th percentile of RNLS RNAseq values, HR = 0.969, *p* = 0.876; for cases representing the lower 75th percentile of RNLS RNAseq values, HR = 0.702, *p* = 0.0009. When generating the Electrostatic CSs with the RP220 AA sequence, for cases representing the upper 25th percentile of RNLS RNAseq values, HR = 0.687, *p* = 0.0023; for cases representing the lower 75th percentile of RNLS RNAseq values, HR = 1.07, *p* = 0.713.

To determine whether RNLS expression levels were relevant to the correlation of the Electrostatic CSs with OS probabilities, we first assessed the range of RNLS expression levels in the SKCM dataset (Figure 3A). Based on the range indicated, we next identified differences in OS probabilities for the upper 25th percentile and lower 75th percentiles and found that an elevated RNLS mRNA expression is associated with worse OS probability (Figure 3B). The observation is consistent with prior reports of *in vitro* and *in vivo* findings [2]. Next, we determined that a survival distinction in OS probabilities for Electrostatic CSs calculated from TCR CDR3-RNLS AA pairs (with the TCR CDR3s recovered from WXS files) can be observed only for cases representing the lower 75th percentile of RNLS expression (Figure 3C). This result was consistent for the assessments of both the full-length RNLS AA sequence and the RP220 peptide (Figure 3C).

### RNLS epitope mapping

**Figure 4:**
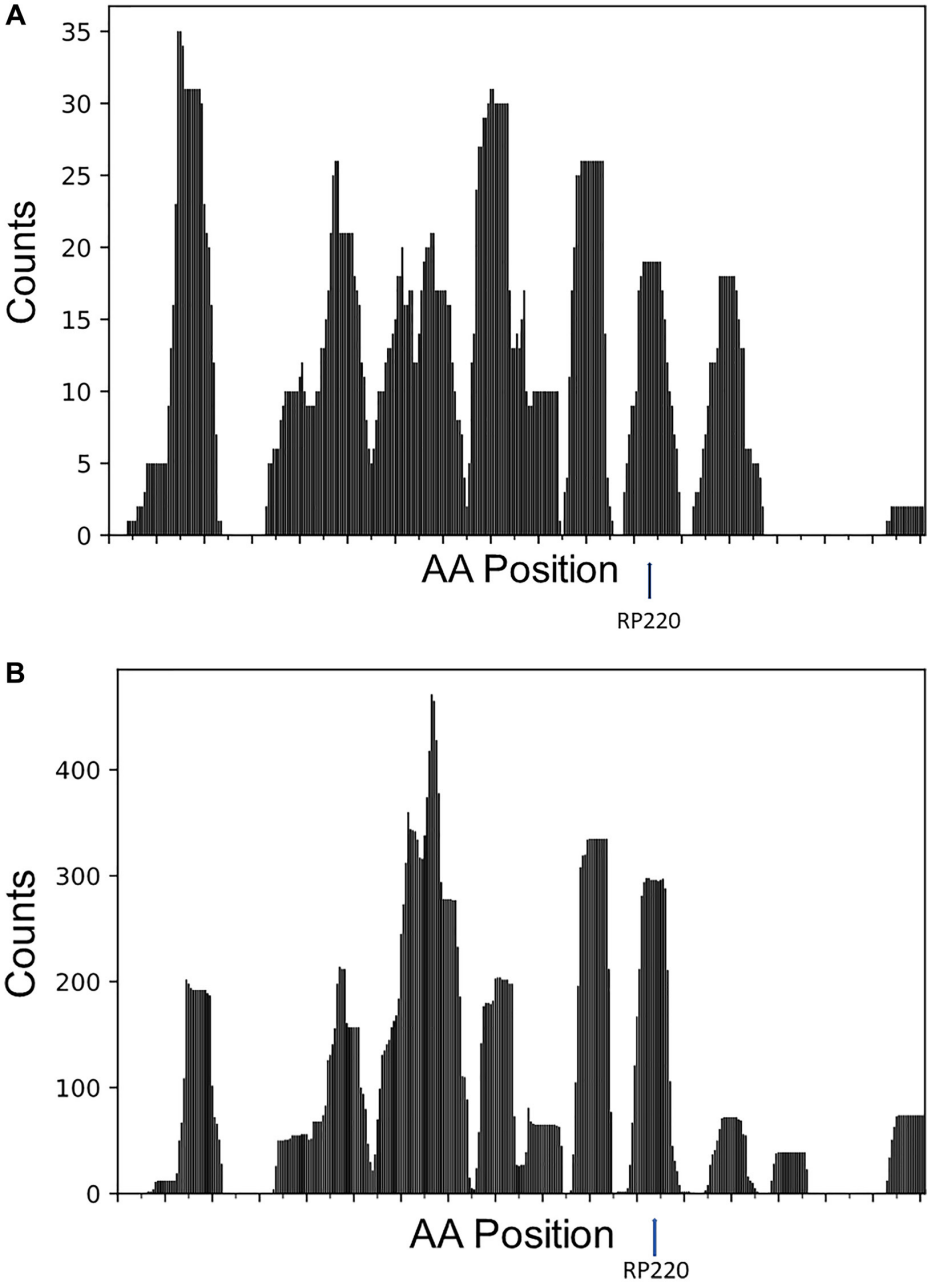
Specific RNLS epitopes contribute to the highest Electrostatic CSs, based on the TCR CDR3s recovered from the TCGA-SKCM WXS and RNAseq files. The histograms display the distribution frequency of RNLS amino acid (AA) residues that overlap the TCR CDR3s that represented the highest Electrostatic CSs (Methods). The X-axis represents the AA position for RNLS; the Y-axis represents the number of counts, where each count corresponds to the number of times an AA in RNLS overlaps a CDR3. The arrow denotes the peak distribution frequency corresponding to RP220. The epitope maps were generated using TCR CDR3s obtained from (**A**) the TCGA-SKCM WXS files and (**B**) the TCGA-SKCM RNAseq files.

Antibodies to RNLS-based peptide, RP220, are potent inhibitors of cancer cell growth in cultured cells and *in vivo* [1–3]. To identify other potentially antigenic RNLS peptides, we mapped the AA sequences of the RNLS protein with the highest complementarity score to the CDR3s assayed (Figure 4) as detailed in Methods. The histograms (Figure 4) illustrate the distribution of residues most frequently interacting with the TRA or TRB CDR3s, based on high Electrostatic CSs for TCR CDR3s obtained from TCGA-SKCM WXS files (Figure 4A) and TCGA-SKCM RNAseq files (Figure 4B) (CS >3 in both cases). Our results demonstrate that the epitopes recognized by TRA and TRB CDR3s with high Electrostatic CS are clustered in specific regions of the RNLS protein, including the RP220 peptide.

## DISCUSSION

In this study, we asked whether the RNLS protein could potentially be a tumor antigen by examining chemical complementarity between melanoma tumor-resident TCR CDR3s and the AA sequence of RNLS. We found a correlation between increasing complementarity of TCR CDR3s to RNLS (and the RP220 peptide) and improved overall survival (OS) outcomes. To our knowledge, this is the first report of associations of CDR3-antigen interactions generated in silico that supports prior data generated *in vitro* and *in vivo*, as in the case of the monoclonal antibodies that target RP220 and potently inhibit cancer growth in models of both melanoma and pancreatic cancer [2, 3, 9].

The results suggest that there could be biologically relevant antigenic interaction between RNLS epitopes and T-cell receptors (TCRs). We hypothesize that RNLS protein could be recognized by TCRs, leading to local immune responses against melanoma, similar to what we have previously demonstrated with wildtype cancer antigens in the melanoma and glioblastoma settings [5, 10]. However, further research is needed to investigate the specific mechanisms and functional significance of the interaction between the RNLS protein and the adaptive immune system in melanoma.

Gene expression analyses were performed to gain further insights into the potential functional implications of TCR complementarity. We demonstrated that several genes associated with T cell activation show increased local tumor mRNA expression as the CS between the TCRs and the RNLS AAs increases. This suggests that the observed association between TCR complementarity and OS outcomes may be related to enhanced T-cell activity and anti-tumor immune responses.

Additionally, we investigated the impact of RNLS expression levels on the association between TCR complementarity scores and OS probabilities and found that the association was more robust in cases with low RNLS expression levels. This implies that high TCR complementarity to RNLS may be particularly beneficial in tumors with lower levels of RNLS expression, where the inhibitory effects of RNLS on antitumor immune response might be limited. Additionally, there could be enhanced recognition of RNLS as a potential antigen in settings with low RNLS expression in the tumor. It is possible that enhanced T-cell-mediated tumor immunity in the setting of low RNLS extends beyond melanoma. Outcomes of quantifying RNLS expression by quantitative immunohistochemistry for pancreatic adenocarcinoma [9] and colon cancer have shown that survival is inversely related to tissue levels. Inhibitory RNLS antibodies markedly decreases tumor RNLS expression in murine melanoma models, and they may enhance T-cell-mediated tumor immunity against tumors expressing high levels of RNLS.

In sum, our findings suggest a potential role for RNLS as an antigen for TCRs in the melanoma setting. The observed correlation between TCR complementarity to RNLS AAs and improved OS outcomes supports the hypothesis that T-cell responses against RNLS may contribute to antitumor immunity. These findings support further exploration of RNLS as a valuable target for immunotherapy and vaccine design.

## MATERIALS AND METHODS

### Recovery of the adaptive immune receptor (IR) reads from tumor exome (WXS) files and RNAseq files

The recovery of adaptive IR reads from WXS files has been described and extensively benchmarked [11–14]. Briefly the WXS files of the TCGA-SKCM dataset (phs000178), representing 469 primary and metastatic cases, were obtained from the National Cancer Institute Genomic Data Commons (GDC) Portal (https://gdc.cancer.gov/) via database of genotypes and phenotypes (dbGaP) project approval number 6300. TCR recombination reads from the TCGA-SKCM RNAseq files were obtained via ref. [6]. WXS primary tumor files representing 72 melanoma cases from the Moffitt Cancer Center were obtained under the Moffitt Cancer Center scientific protocol number 18129. An adaptive IR read was used in the analyses below only in cases of a verifiable V- and J-gene segment within one sequencing read. The software for extracting the recombination reads from genomics files is available at: https://github.com/bchobrut-USF/blanck_group (with readme files); https://github.com/kcios/2021 [15]; and a dockerized version is at https://hub.docker.com/r/bchobrut/vdj (also including a readme file). Data representing the adaptive IR recombination reads from the TCGA-SKCM and Moffitt Cancer Center WXS files used in this report are in supporting online material (SOM) Supplementary Tables 1 and 2, respectively. (Note: SOM tables and figures can be downloaded from: https://usf.box.com/s/qoxgvgrxmb2ch0a31av84ysjvkchcg1x). The data representing the adaptive IR recombination reads from the TCGA-SKCM RNAseq files were obtained from ref. [6].

### Calculation of chemical complementarity scores (CS)

The chemical CS calculations are based on our previous work [7, 16–18]. Briefly, the CDR3 AA sequence and the candidate antigen sequence (full-length human RNLS AA sequence or the biologically active RNLS peptide, RP220, representing AA 220 to 239 of human RNLS protein [19], were aligned, and an assessment of chemical affinity was made based on the juxtaposition of the CDR3 and candidate antigen AA sequences. For example, a negatively charged R group in the candidate antigen directly across from a positively charged R group in the CDR3 would have a relatively high value. If the two AAs are adjacent but not directly aligned, the value contribution to the final score of the alignment correspondingly decreases. The complete set of mathematical details are available in ref. [7]. A CS is thus calculated for the entire CDR3-candidate epitope alignment. Then, there is a one-AA shift in the alignment, and the CS is recalculated, with the highest CS value produced by all CDR3-candidate antigen alignments retained for subsequent analyses. The entire software package for this “sliding window” assessment process is available at https://github.com/bchobrut/brca_swcs, along with a readme file, and the assessments were facilitated by the web tool at adaptivematch.com. The web tool can calculate the CDR3-candidate antigen CSs and sort the cases into upper and lower 50th percentiles based on the maximal CDR3-candidate antigen CS. The adaptivematch.com web tool then outputs whether there is a statistically significant survival probability distinction representing the upper and lower percentile groups. Example input and output files for adaptivematch.com are provided in the SOM (Supplementary Table 3, TCR CDR3 input file; Supplementary Table 4, candidate antigen input file; Supplementary Table 5, OS data input file; Supplementary Table 6, summary output file; Supplementary Table 7, raw CS output file). Further details regarding file formats can be found at https://adaptivematch.com/.

### Cox proportional hazards model

Clinical data were downloaded from cbioportal.org for TCGA-SKCM (pancancer). Clinical information for the Moffitt Cancer Center melanoma cases were obtained under Moffitt Cancer Center protocol number 18129. Relative hazard ratios (HRs) for death were estimated with a univariate Cox proportional hazards model using the lifelines Python package (https://doi.org/10.21105/joss.01317) [20]. Forrest plots of individual HRs and their 95% confidence intervals were generated using the Python Matplotlib package [21].

### Gene expression analyses and associated survival analyses

Gene expression data (RNAseq RSEM V2 values) for a previously described and applied panel of immune signature genes [5, 22] (Supplementary Table 8) were downloaded from cbioportal.org. Pearson’s correlation analyses for the gene expression levels and the CSs for the TCR CDR3s and candidate antigens was performed using the Python SciPy package. Pearson’s correlation data were then further processed using Graphpad Prism software version 7.0, to generate histograms of the Pearson’s coefficients for each gene assessed (Supplementary Figure 1). The Bonferroni correction was applied to adjust the threshold for statistical significance, dividing the alpha level (e.g., 0.05) by the number of tests performed. Only test results with a *p*-value below this corrected threshold were considered statistically significant. Kaplan-Meier (KM) plots for survival analyses comparing cases representing the upper 50th percentile of expression levels for the indicated genes versus cases representing the lower 50th percentile was generated using the Gene Expression Profiling Interactive Analysis (GEPIA) web tool [23]. The generated plots were converted to PDFs via the web tool option, and the plots in the figures of this report were taken from those PDFs.

### Epitope mapping of the RNLS AA sequence

TRA and TRB CDR3 Electrostatic complementarity scores (CSs) with a minimum CS of 3.0 or greater obtained from a scan of the entire RNLS AA sequence were used to generate an initial collection of CDR3-peptide pairs. For each TRA and TRB CDR3-epitope peptide available, a count value of 1 was assigned to each AA in such RNLS peptides. Thus, the resulting epitope map displays a histogram of the frequency of each AA residue that contributes to high CSs for multiple CDR3-RNLS peptide pairs. The Python script that generated the epitope map histogram can be found in Supplementary Table 9.

## SUPPLEMENTARY MATERIALS





## Abbreviations

AA: amino acid
CDR3: complementarity-determining region-3
CS: (chemical) complementarity score
dbGaP: database of genotypes and phenotypes
GDC: genomic data commons
IR: (adaptive) immune receptor
KM: Kaplan-Meier
OS: overall survival
RNLS: renalase gene HUGO symbol
SKCM: Skin Cutaneous Melanoma dataset
SOM: supporting online material
TCR: T-cell receptor
TCGA: the cancer genome atlas
WXS: whole exome sequence
