# Supplementary material for: Chemical complementarity of tumor resident, T-cell receptor CDR3s and renalase-1 correlates with increased melanoma survival
Source: Oncotarget. 2024 Aug 5;15:550–61. doi: 10.18632/oncotarget.28633 (PMC11299663; doi:10.18632/oncotarget.28633)
Supplement: Supplementary file 1 [file oncotarget-15-28633-s001.pdf]

Chemical complementarity of tumor resident, T-cell receptor CDR3s and renalase-1 correlates with increased melanoma survival

SUPPLEMENTARY MATERIALS

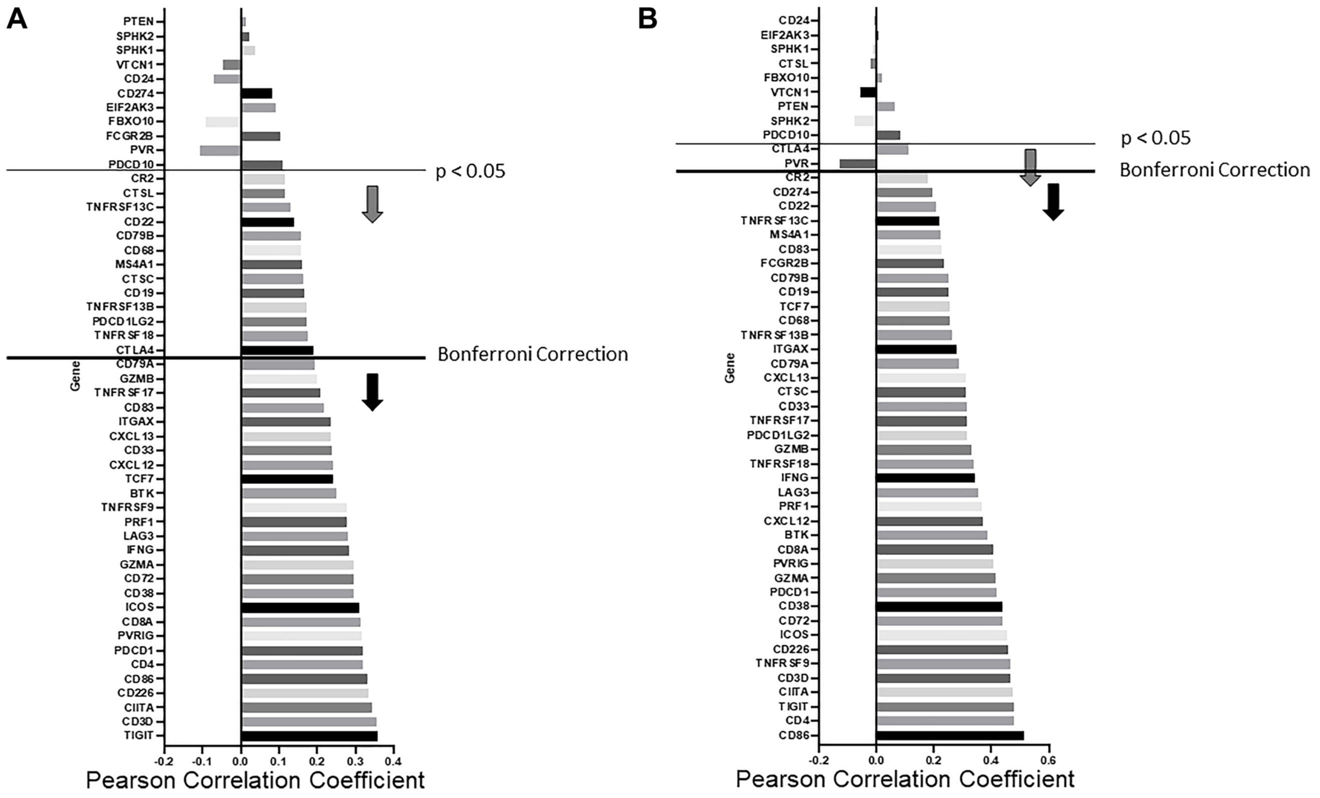

Supplementary Figure 1: Pearson Correlation analysis of TCR-related gene expression and electrostatic RNLS complementarity to TRA and TRB CDR3s. Histogram showing the Pearson correlation coefficients between gene expression and electrostatic complementarity score for the indicated genes. The X-axis represents the Pearson correlation coefficients; −1 would represent a perfect negative correlation) and +1 a perfect positive correlation. The Y-axis represents the genes derived from the immune signature gene panel. Genes below the thin line have Pearson correlation coefficients with $p < 0.05$ (grey arrow). Genes below the thick ffne have. Pearson correlation coefficients corrected for Bonferroni correction for multiple comparfsons correction, $p < 0.00098$ (black arrow) (**A**) Pearson correlation analysis with WXS-recovered electrostatic complementarlty scores in TCGA-SKCM samples, 27 of 51 genes assayed nave significant positive Pearson correlation coefficients when using Bonferroni correction. (**B**) Pearson correlation analysis with RNAseq-recover:ed electrostatic complementarity scores in TCGA-SKCM samples 40 of 51 genes assayed have significant positive Pearson correlation coefficients when using Bonferroni correction.

Supplementary Table 1: SKCM, WXS based CDR3 recoveries. See Supplementary Table 1

Supplementary Table 2: Moffitt Cancer Center WXS file derived, TRA and TRB recombination read data. See Supplementary Table 2

Supplementary Table 3: TCR CDR3 input file, RNAseq-based CDR3s for SKCM. See Supplementary Table 3

Supplementary Table 4: Candidate antigen input file

antigen	antigen_sequence
RP-220	CIRFVSIDNKKRNIESSEIG
RNLS-1	MAQVLIVGAGMTGSLCAALLRRQTSGPLYLAVWDKAEDSGGRMTTACSPHNPQCT ADLGAQYITCTPHYAKKHQRFYDELLAYGVLRPLSSPIEGMVMKEGDCNFVAPQGIS SIIKHYLKESGAEVYFRHRVTQINLRDDKWEVSKQTGSPEQFDLIVLTMPVPEILQLQ GDITTLISECQRQQLEAVSYSSRYALGLFYEAGTKIDVPWAGQYITSNPCIRFVSIDNK KRNIESSEIGPSLVIHTTVPFGVTYLEHSIEDVQELVFQQLENILPGLPQPIATKCQKWR HSQVTNAAANCPGQMTLHHKPFLACGGDGFTQSNFDGCITSALCVLEALKNYI

Supplementary Table 5: SKCM survival input file. See Supplementary Table 5

Supplementary Table 6: SKCM survival output file. See Supplementary Table 6

Supplementary Table 7: Raw complementarity score outputs. See Supplementary Table 7

Supplementary Table 8: Immune signature genes

CD274
CD33
CD3D
CD4
CD68
CD83
CD8A
CIITA
CTLA4
CTSL
CXCL12
CXCL13
FBXO10
ICOS
IFNG
ITGAX
PDCD1
PDCD10
TNFRSF18
VTCN1
PTEN

CD19
CD22
CD24
CD38
CD72
CD79A
CD79B
CD86
CR2
FCGR2B
MS4A1
TNFRSF13B
TNFRSF13C
TNFRSF17
PDCD1LG2
LAG3
TNFRSF9
GZMA
GZMB
CTSC
SPHK1
SPHK2
PRF1

Supplementary Table 9: Python script that generated the epitope map histogram. See Supplementary Table 9
